# Correction: König et al. Cefiderocol in Critically Ill Patients with Multi-Drug Resistant Pathogens: Real-Life Data on Pharmacokinetics and Microbiological Surveillance. *Antibiotics* 2021, *10*, 649

**DOI:** 10.3390/antibiotics10101230

**Published:** 2021-10-09

**Authors:** Christina König, Anna Both, Holger Rohde, Stefan Kluge, Otto R. Frey, Anka C. Röhr, Dominic Wichmann

**Affiliations:** 1 Department of Intensive Care Medicine, University Medical Center Hamburg-Eppendorf, 20251 Hamburg, Germany; ch.koenig@uke.de (C.K.); s.kluge@uke.de (S.K.); 2 Department of Pharmacy, University Medical Center Hamburg-Eppendorf, 20251 Hamburg, Germany; 3 Department of Microbiology, University Medical Center Hamburg-Eppendorf, Virology and Hygiene, 20251 Hamburg, Germany; a.both@uke.de (A.B.); h.rohde@uke.de (H.R.); 4 Department of Pharmacy, General Hospital of Heidenheim, 89522 Heidenheim, Germany; otto.frey@kliniken-heidenheim.de (O.R.F.); anka.roehr@kliniken-heidenheim.de (A.C.R.)

## 1. Text Correction

1.1 Based on the initial data from Table 1 there were errors in the original article [1]: “Five patients were included into the study, four were men, the median age was 53 years (range 35 to 76).” has been corrected to *Results, Individual Patients, and Paragraph 1*:

“Five patients were included into the study, four were men, the median age was 55 years (range 41 to 76).”

1.2 In addition, the statement regarding patient #1: “Therapy with cefiderocol accompanied by TDM was initiated with a dose of 2 g eight hourly”, has been corrected to: *Results, Individual Patients*, *Paragraph 2, Patient #1*:

“Therapy with cefiderocol accompanied by TDM and dose adaptions was initiated with a dose of 1 g eight hourly administered in prolonged infusion mode according to the manufactures’ instructions. “

1.3 The statements in patient #2: “Therapy with cefiderocol adapted for renal insufficiency accompanied by TDM was initiated with a dose of 1 g eight hourly administered in prolonged infusion mode.”, has been corrected to: *Results, Individual Patients*, *Paragraph 2, Patient #2:*

“Therapy with cefiderocol adapted for renal insufficiency accompanied by TDM was initiated with a dose of 1 g eight hourly administered in prolonged infusion mode after a loading dose of 3 × 2 g for 24 h.”

1.4 Furthermore, the statement regarding patient #3: “On day 4 CRRT was initiated due to acute renal failure.” is incorrect and has been deleted.

1.5 The sentence: “After the first dose the treatment was extended by cytokine adsorber therapy with (CytoSorb^®^, Monmouth Junction, NJ, USA).”, has been corrected to: *Results, Individual Patient, Paragraph 2, Patient #5:*

“After the first dose the treatment was extended by cytokine adsorber therapy (CytoSorb^®^, Monmouth Junction, NJ, USA).”

1.6 The statement: “Samples for TDM were collected on day two of cefiderocol treatment initiation and repeated with occurrence of either dose adjustment or alteration in renal function including initiation of extracorporeal organ support.”, has been corrected to: *Materials and Methods, 4.3 Cefiderocol Quantification:*

“Samples for TDM were collected from the beginning of cefiderocol treatment initiation and repeated with occurrence of either dose adjustment or alteration in renal function including initiation of extracorporeal organ support. “

1.7 The statement: “These findings, confirm the high variability in drug elimination in the vulnerable cohort of critically ill patients.”, has been corrected to: *3. Discussion:*

“These findings do confirm the high variability in drug elimination in the vulnerable cohort of critically ill patients.”

The authors apologize for any inconvenience caused and state that the scientific conclusions are unaffected. The original article has been updated.

## 2. Error in Table 1

In the original article, there were mistakes in Table 1 as published: “The age of patient #1 was stated as 35 years and the cefiderocol trough levels of patient #1 were described to be drawn on day 2 and 4. The age of patient #2 was stated as 60 years and the cefiderocol trough levels were stated to be drawn on day 1. The age of patient #5 was stated as 55 years.”

The corrected Table 1 appears below.

The authors apologize for any inconvenience caused and state that the scientific conclusions are unaffected. The original article has been updated.

## Figures and Tables

**Table 1:** Demographic data, extracorporeal therapies and microbiological data.

Case	Sex	Age [Years]	Weight [kg]	CKD-EPI eGFR (mL/min/1.72 m^2^) or Mode of Renal Replacement Therapy	Cefiderocol Dose [mg/24 h]	Cefiderocol Through Level [mg/L]	Focus of Infection	SOFA/SAPS	Medical History	Outcome Related to the Event	Pathogen Isolated	MIC [mg/L]	
												First Available Isolate	Last Available Isolate
1	m	41	75	CVVHD DFR 2.0 L/min *	2000	25.2 (day 4)	CAP	11/49	PAH; interstitial lung disease; chronic renal impairment	CC + MC	*P. aeruginosa*	0.5	0.25
				CVVHD DFR 2.0 L/min *	2000	32.0 (day 5)							
2	f	69	60	67	3000	70 (day 3)	HAP	4/33	Esophagectomy, IPA, mediastinitis	MC	*P. aeruginosa*	0.25	n. a.
				22	2000	49 (day 7)							
3	m	76	85	84	6000	36.8 (day 1)	HAP; Primary Sepsis	9/49	COVID-19; ARDS, ECMO	MC	*A. baumannii*	0.25	0.25
				85	6000	43.2 (day 3)							
				81	6000	59.5 (day 4)							
4	m	53	60	28 CVVHD DFR 2.4 L/min*	6000 3000	42 (day 1) >100 (day 3)	Primary Sepsis	14/68	ASCT; GvHD c/I; invasive mold infection; HHV-6 encephalitis; BKV-cystitis	died	*P. aeruginosa*	0.125	16
5	m	50	90	CVVHD DFR 2.4 L/min * + CytoSorb^®^ **	6000	26 (day 1) 13 (day 2)	HAP	19/59	COVID-19; ARDS, ECMO	died	*A. baumannii*	0.25	n.a.

ARDS = acute respiratory distress syndrome; ASCT = allogenic stem cell transplantation; BKV = human papilloma virus; CAP = community acquired pneumonia; CC = clinical cure; COVID-19 = coronavirus disease 19; CVVHD = continuous venovenous haemodialysis; DFR= dialysate flow rate; eGFR= estimated glomerular filtration rate calculated by CKD-EPI; f = female; GvHD c/l = graft versus host disease cutaneous/lung; HAP = hospital acquired pneumonia; HHV-6= human herpes virus 6; IPA = invasive pulmonary aspergillosis; m = male; MC = microbiological cure; MIC = minimal inhibitory concentration; n.a. = not applicable; SAPS = simplified acute physiology score; SOFA = sequential organ failure assessment; PAH = pulmonary hypertension; * used with the Ultraflux V600S filter with a 1.4 m^2^ surface area (Fresenius Medical Care, Germany); ** installed in series with the CVVHD, with a blood flow of 200 mL/min (CytoSorbents Corporation, Monmouth Junction, NJ, USA).
